# A comparison of long‐term clinical outcomes between percutaneous coronary intervention (PCI) and medical therapy in patients with chronic total occlusion in noninfarct‐related artery after PCI of acute myocardial infarction

**DOI:** 10.1002/clc.23771

**Published:** 2022-01-06

**Authors:** Qing Qin, Lu Chen, Lei Ge, Juying Qian, Jianying Ma, Junbo Ge

**Affiliations:** ^1^ Department of Cardiology, Zhongshan Hospital, Fudan University Shanghai Institute of Cardiovascular Disease Shanghai China

**Keywords:** acute myocardial infarction, chronic total occlusion, percutaneous coronary intervention

## Abstract

**Background:**

Chronic total occlusion (CTO) in a noninfarct‐related artery (IRA) is one of the risk factors for mortality after acute myocardial infarction (AMI). However, there are limited data comparing the long‐term outcomes of patients undergoing percutaneous coronary intervention (PCI) with patients having medical therapy (MT) in CTO lesion after AMI PCI.

**Methods:**

We retrospectively enrolled 330 patients (successful CTO PCI in 166 patients, failed CTO PCI in 32 patients, MT in 132 patients) with non‐IRA CTO from a total of 4372 patients who underwent PCI after AMI in our center. Propensity score matching (PSM) was used to adjust for baseline differences.

**Results:**

The primary analysis is based on the intention‐to‐treat population. During a median follow‐up period of 946 days, patients in the PCI group (*n* = 198) had significantly higher cardiac death‐free survival (96.6% vs. 82.8%, *p* = .004) compared with patients in MT group (*n* = 132). However, no significant difference in the occurrence of cardiac death was observed after PSM. The analysis based on the per‐protocol population demonstrated significantly higher cardiac death‐free survival in the successful CTO PCI group (*n* = 166) compared with the occluded CTO group (*n* = 164) both before and after PSM. In subgroup analysis, successful CTO PCI was associated with less cardiac death in patients over 65 years old, with LVEF < 50%, left anterior descending (LAD) IRA, and non‐LAD CTO lesion compared with occluded CTO group.

**Conclusions:**

Patients undergoing successful revascularization of non‐IRA CTO after AMI might have a better long‐term prognosis. Moreover, patients with LVEF < 50% may benefit from successful non‐IRA CTO PCI after AMI.

## 1 INTRODUCTION

In the contemporary practice, among patients with acute myocardial infarction (AMI) undergoing percutaneous coronary intervention (PCI), around 50%–60% present with multivessel disease (MVD)^1^, ^2^ and 8%–13% have concurrent chronic total occlusion (CTO) lesion.^3^, ^4^ Previous studies have shown that AMI with MVD was associated with worse clinical outcomes and complete revascularization in these patients will lead to reduced adverse cardiovascular events.^3^, ^5^ MVD with a coexisting CTO lesion in a noninfarct‐related artery(non‐IRA) is an independent predictor for long‐term mortality in AMI patients,^3^, ^4^, ^6^ and one study even reported that the presence of CTO alone but not MVD is associated with long‐term mortality,^7^ indicating the strong association of CTO lesion with cardiac mortality in these patients.^8^ However, whether revascularization of CTO lesion in non‐IRA will lead to improved clinical outcomes is still controversial. Observational studies^9^, ^10^, ^11^, ^12^ and meta‐analysis^13^, ^14^ favor CTO‐PCI; however, the only randomized trial in this field, EXPLORE (Evaluating Xience and Left Ventricular Function in Percutaneous Coronary Intervention on Occlusions After ST‐Elevation Myocardial Infarction) trial failed to confirm the benefit of staged PCI of non‐IRA CTO in terms of major adverse cardiovascular events (MACEs).^15^ Therefore, this study aimed to evaluate the long‐term impact of CTO revascularization in AMI patients after IRA PCI in the real world.

## 2 METHODS

### 2.1 Patient population

All consecutive patients diagnosed as AMI (including STEMI and non‐ST segment elevation myocardial infarction [NSTEMI]) and who underwent coronary artery angiography (CAG) in Zhongshan Hospital, Shanghai, China, between July 2011 and July 2019 were retrospectively included in this study. Patients with prior coronary artery bypass graft (CABG) were excluded. We then identified patients treated by successful PCI in IRA and had at least one coexisting non‐IRA CTO in the major epicardial coronary arteries. Patients who died during hospital stay after IRA PCI and patients treated by CABG after PCI were excluded from the study. The study was approved by the institutional review board of Zhongshan Hospital, Fudan University and all patients signed a general informed consent form.

### 2.2 Study definitions and endpoints

AMI was diagnosed according to characteristic clinical symptoms, ECGs changes, cardiac enzyme elevations (Fourth Universal Definition^16^), and was also confirmed by CAG. Periprocedural MIs were not included in the study. IRA was defined as a major coronary artery perfusing an area compatible with the distribution of ST‐segment elevation or depression in the 12‐lead ECG and the typical angiographic image. CTO was defined as thrombolysis in myocardial infarction (TIMI) Grade 0 flow and duration of coronary occlusion ≥3 months. In addition, the typical appearance of a CTO includes angiographically visible mature collaterals and the absence of thrombus or staining at the proximal cap.^17^ Only CTOs of major epicardial coronary arteries (CTO in left anterior descending [LAD], left circumflex coronary artery [LCX], or right coronary artery [RCA]) with estimated vessel diameter ≥ 2.5 mm were included in the study. Technical success was defined as an antegrade TIMI flow grade ≥ 2 in the CTO target vessel with residual stenosis < 30%. After PCI, patients were treated with dual antiplatelet therapy (aspirin and clopidogrel or ticagrelor) and maintained for at least 12 months. The primary clinical endpoint on follow‐up was cardiac death. The secondary clinical endpoint was a major adverse cardiovascular and cerebrovascular event (MACCE), defined as the composite of all‐cause death, stroke, nonfatal MI, and any revascularization. All deaths were considered cardiac unless otherwise documented. Stroke was defined as a new focal neurological deficit lasting >24 h, which was confirmed by neurologists based on both clinical and radiographic criteria.^12^ Any revascularization was defined as a repeat PCI or CABG excluding the planned staged PCIs of any segment of the coronary artery.

### 2.3 Procedures

All patients were treated with 300 mg aspirin and a loading dose of 300 mg of clopidogrel or 180 mg of ticagrelor before the procedure. During the procedure, unfractionated heparin was administered intravenously to achieve a target activated clotting time of 250–350 s. GPIIb/IIIa inhibitors were administered at the operator's discretion. IRA stenting was performed using a drug‐eluting stent (DES). Successful IRA PCI was defined as residual stenosis of the culprit lesion <30% and a TIMI flow grade ≥ 2. When to perform PCI in non‐IRA vessels (CTO or non‐CTO lesion) was left to the operator's discretion, usually within 1 year after IRA PCI. For CTO PCI, the choices of antegrade or retrograde approach and devices used were up to the discretion of the operator. DES was used in successfully recanalized CTO vessels.

### 2.4 Data collection

Demographic, angiographic, procedural, and outcome data were obtained from a review of the catheterization laboratory database and medical chart. Clinical follow‐up data were collected through outpatient visits, telephone interviews, and medical chart reviews.

### 2.5 Statistical analysis

The primary analysis is based on the intention‐to‐treat (ITT) population. All continuous variables were presented as mean ± standard deviation or the median with interquartile range and were compared by Student's *t* test or the Mann–Whitney *U* test, respectively. Categorical variables were presented as counts and percentages and were compared by *χ* ^2^ test (or Fisher's exact test when appropriate). To adjust for any potential confounders, propensity score matching (PSM) analysis was performed using the logistic regression model. Variables that could be of potential relevance to the endpoints, including age, male, hypertension, diabetes, dyslipidemia, current smoking, previous MI, previous PCI, IRA, location of CTO, and left ventricular ejection fraction (LVEF), were used. Matching was performed via a 1:1 matching protocol using the nearest neighbor matching algorithm, with a caliper width equal to 0.05 of the standard deviation of the propensity score. The covariate balance of the matched cohort was assessed using the standardized mean difference (SMD).^18^ The C‐statistics for PSM was 0.757 in the ITT population. Survival curves were plotted using the Kaplan–Meier method, and comparisons between groups were done using the log‐rank test. The Cox proportional hazards model was used to identify the independent predictors of cardiac death. The candidate variables for the model were selected based on significant univariate analysis. Prespecified subgroup analyses were performed for the primary endpoint according to the following variables: age, gender, diagnosis, diabetes, LVEF, IRA, and CTO location. All analyses were performed using SPSS, Version 20.0 (IBM Corporation), and a *p* < .05 was considered statistically significant.

## 3 RESULTS

### 3.1 Baseline characteristics in ITT population

**Figure 1:**
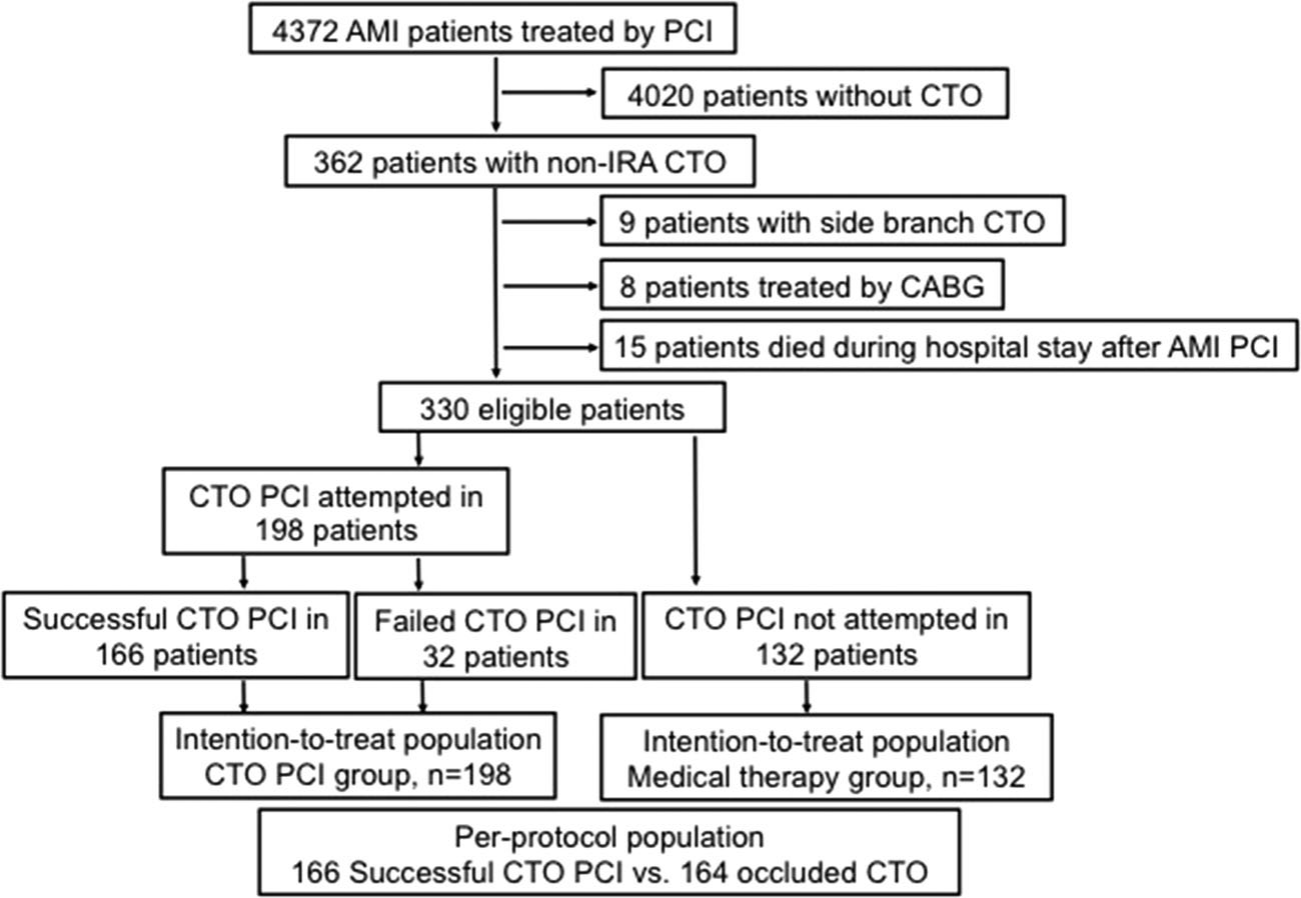
Flow chart of the study. AMI, acute myocardial infarction; CABG, coronary artery bypass graft; CTO, chronic total occlusion; non‐IRA, non‐infarct related artery; PCI, percutaneous coronary intervention

Among 4372 patients who were diagnosed with AMI and treated by IRA PCI during the study period, we identified 362 eligible patients who had non‐IRA CTOs. Of these patients, eight were excluded as the CTO lesions were treated by CABG, 15 patients were excluded because they died during hospital stay after IRA PCI, and nine patients were excluded because the CTO lesions were not located in major epicardial coronary arteries. Finally, 330 patients who were treated by either PCI (*n* = 198) or MT (*n* = 132) for non‐IRA CTOs were included in the study (Figure 1).

The baseline, angiographic, and procedural characteristics of patients during IRA PCI are listed in Table 1. The patients in the MT group were older, more likely to be diagnosed as STEMI, had lower estimated glomerular filtration rate (eGFR), higher peak troponin T, and creatinine kinase (CK)‐MB level during AMI compared with patients in the PCI group. Furthermore, in MT group, the involvement of LAD coronary artery as IRA (53.8% vs. 38.4%, *p* = .006) and LCX as CTO vessel (45.5% vs. 29.8%, *p* = .004) was more frequent than in PCI group, and thus the involvement of LAD as CTO vessel was less frequent (14.4% vs. 39.4%, *p* < .001). The baseline, angiographic, and procedural characteristics of the two groups were balanced after PSM (108 pairs).

**Table 1:** Baseline and procedural characteristics during AMI PCI in the intention‐to‐treat population

	All patients			Standardized	PSM patients		*p* value	Standardized
	PCI (*n* = 198)	MT (*n* = 132)	*p* value	difference	PCI (*n* = 108)	MT (*n* = 108)		difference
Male (%)	180 (90.9)	117 (88.6)	.500	0.076	97 (89.8)	97 (89.8)	>.999	<0.001
Age (years)	60.9 ± 11.8	64.4 ± 12.4	.011	−0.293	61.7 ± 12.4	62.5 ± 12.0	.636	−0.066
Hypertension (%)	135 (68.2)	86 (65.2)	.566	0.064	73 (67.6)	74 (68.5)	.884	−0.019
Diabetes (%)	79 (39.9)	40 (30.3)	.075	0.202	37 (34.3)	35 (32.4)	.773	0.040
Insulin (%)	14 (7.1)	12 (9.1)	.505	−0.073	4 (3.7)	11 (10.2)	.095	−0.258
Dyslipidemia (%)	16 (8.1)	11 (8.3)	.935	−0.007	12 (11.1)	7 (6.5)	.230	0.163
Current smoking (%)	65 (32.8)	53 (40.2)	.174	−0.154	40 (37.0)	40 (37.0)	>.999	<0.001
Previous MI (%)	47 (23.7)	21 (15.9)	.085	0.197	23 (21.3)	19 (17.6)	.492	0.094
Previous PCI (%)	39 (19.7)	24 (18.2)	.732	0.038	20 (18.5)	21 (19.4)	.862	−0.022
Diagnosis								
STEMI (%)	82 (41.4)	72 (54.5)	.019	−0.262	46 (42.6)	53 (49.1)	.339	−0.131
Lab test								
TC (mmol/L)	4.4 ± 1.1	4.5 ± 1.2	.384	−0.087	4.5 ± 1.2	4.4 ± 1.2	.730	0.083
TG (mmol/L)	2.0 ± 1.4	1.8 ± 1.3	.269	0.148	2.0 ± 1.3	1.9 ± 1.4	.747	0.074
LDL‐C (mmol/L)	2.5 ± 1.1	2.7 ± 1.1	.214	−0.182	2.7 ± 1.1	2.7 ± 1.1	.946	<0.001
HDL‐C (mmol/L)	1.0 ± 0.3	1.0 ± 0.3	.286	<0.001	1.0 ± 0.3	1.0 ± 0.3	.483	<0.001
eGFR (ml/min/1.73 m2)	85.3 ± 24.2	78.7 ± 30.5	.029	0.240	85.3 ± 26.7	80.1 ± 31.2	.191	0.179
HbA1c (%)	6.8 ± 1.6	6.8 ± 1.8	.949	<0.001	6.7 ± 1.6	6.8 ± 1.8	.540	−0.059
LVEF (%)	52.9 ± 10.0	51.6 ± 10.6	.239	0.126	53.4 ± 9.5	52.3 ± 10.3	.420	0.111
LVEF < 50%	69 (36.5)	54 (40.9)	.425	−0.090	36 (33.3)	40 (37.0)	.568	−0.078
Infarct‐related artery								
LM (%)	1 (0.5)	0	>.999	‐	0	0	‐	‐
LAD (%)	76 (38.4)	71 (53.8)	.006	−0.313	53 (49.1)	58 (53.7)	.496	−0.092
LCX (%)	48 (24.2)	23 (17.4)	.140	0.168	19 (17.6)	18 (16.7)	.857	0.024
RCA (%)	74 (37.4)	42 (31.8)	.300	0.118	36 (33.3)	32 (29.6)	.558	0.080
In stent thrombosis (%)	5 (2.5)	6 (4.5)	.358	−0.109	4 (3.7)	5 (4.6)	>.999	−0.045
Location of CTO								
LAD (%)	78 (39.4)	19 (14.4)	<.001	0.596	21 (19.4)	17 (15.7)	.475	0.097
LCX (%)	59 (29.8)	60 (45.5)	.004	−0.326	40 (37.0)	45 (41.7)	.486	−0.096
RCA (%)	72 (36.4)	54 (40.9)	.405	−0.093	47 (43.5)	47 (43.5)	>.999	<0.001
In stent CTO (%)	7 (3.5)	8 (6.1)	.281	−0.122	5 (4.6)	6 (5.6)	.757	−0.045
IABP use (%)	3 (1.5)	1 (0.8)	.652	0.066	1 (0.9)	1 (0.9)	>.999	<0.001
Peak troponin T (ng/ml)	0.58	1.29	.003	−0.320	0.62	1.29	.050	−0.236
	(0.18–2.23)	(0.26–5.23)			(0.1–2.67)	(0.24–4.39)		
Peak creatinine kinase (U/L)	23 (15–66)	34 (18–144)	.023	−0.254	23 (14–71)	30 (17–131)	.105	−0.224
Stents/patient	1.6 ± 0.7	1.5 ± 0.7	.267	0.143	1.4 ± 0.7	1.4 ± 0.7	>.999	<0.001
Average stent diameter (mm)	3.1 ± 0.4	3.0 ± 0.4	.468	0.250	3.1 ± 0.4	3.1 ± 0.4	.850	<0.001
Total stent length (mm)	46.5 ± 24.6	43.2 ± 23.2	.236	0.138	42.3 ± 22.4	42.4 ± 22.0	.982	−0.005

Abbreviations: AMI, acute myocardial infarction; CTO, chronic total occlusion; eGFR, estimated glomerular filtration rate; HbA1c, hemoglobin A1c; HDL‐C, high‐density lipoprotein cholesterol; IABP, intra‐aortic balloon pump; LAD, left anterior descending coronary artery; LCX, left circumflex coronary artery; LDL‐C, low‐density lipoprotein cholesterol; LM, left main coronary artery; LVEF, left ventricular ejection fraction; MI, myocardial infarction; MT, medical therapy; NSTEMI, non‐ST segment elevation myocardial infarction; PCI, percutaneous coronary intervention; PSM, propensity score matching; RCA, right coronary artery; STEMI, ST‐segment elevation myocardial infarction; TC, total cholesterol; TG, triglyceride.

### 3.2 Long‐term clinical outcomes in ITT population

**Figure 2:**
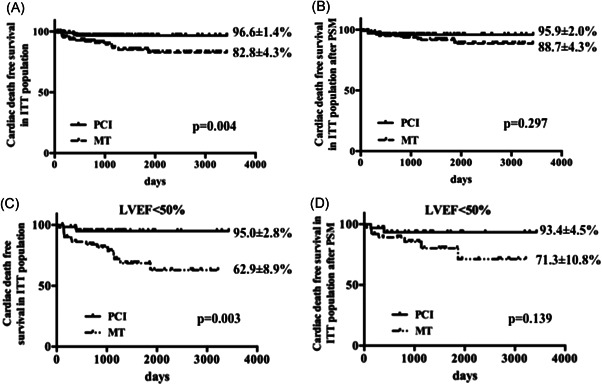
Kaplan–Meier analysis of primary endpoints in ITT population. (A) In the overall population; (B) after PSM; (C) in patients with LVEF < 50%; (D) in patients with LVEF < 50% after PSM. ITT, intention‐to‐treat; LVEF, left ventricular ejection fraction; MT, medical therapy; PCI, percutaneous coronary intervention; PSM, propensity score matching

Clinical outcomes in the entire cohort and PSM groups are presented in Table 2 and Figure 2A,B. During a median follow‐up duration of 946 days (interquartile range: 562–1678 days), patients who underwent PCI had significantly higher cardiac death‐free survival (96.6% vs. 82.8%, *p* = .004) compared with patients in the MT group. However, after PSM, there was no significant difference in the rate of cardiac death‐free survival (95.9% vs. 88.7%, *p* = .297) between PCI and MT groups. The incidence of MI, stroke, revascularization, and MACCE was not significantly different between the two groups both before and after PSM.

**Table 2:** Long‐term clinical outcomes in the intention‐to‐treat population

	All patients		*p* value	PSM patients		*p* value
	PCI (*n* = 198)	MT (*n* = 132)		PCI (*n* = 108)	MT (*n* = 108)	
All‐cause death (%)	10 (5.1)	22 (16.7)	.003	6 (5.6)	14 (13.0)	.111
Cardiac death (%)	6 (3.0)	16 (12.1)	.004	4 (3.7)	8 (7.4)	.297
MI (%)	9 (4.5)	11 (8.3)	.363	4 (3.7)	7 (6.4)	.840
Stroke (%)	3 (1.5)	7 (5.3)	.070	1 (0.9)	6 (5.6)	.073
Revascularization (%)	32 (16.2)	22 (16.7)	.642	19 (17.6)	20 (18.5)	.765
CTO vessel (%)	16 (8.1)	7 (5.3)	‐	9 (8.3)	5 (4.6)	‐
Infarct‐related artery (%)	9 (4.5)	10 (7.6)	‐	6 (5.6)	9 (8.3)	‐
Other (%)	13 (6.6)	12 (9.1)	‐	7 (6.5)	10 (9.3)	‐
MACCE (%)	44 (22.2)	49 (37.1)	.055	25 (23.1)	37 (34.3)	.292

Abbreviations: CTO, chronic total occlusion; MACCE, major adverse cardiovascular and cerebrovascular events; MI, myocardial infarction; MT, medical therapy; PCI, percutaneous coronary intervention; PSM, propensity score matching.

### 3.3 Subgroup analysis in ITT population

In subgroup analysis, PCI was associated with less cardiac death in patients over 65 years old, without diabetes, with LVEF < 50%, LAD IRA, and non‐LAD CTO lesion compared with MT (Figure S1). In patients with LVEF < 50%, the long‐term cardiac death‐free survival is higher in the PCI group compared with the MT group (Figure 2C). However, there was no significant difference in cardiac death after PSM (Figure 2D). In patients with LVEF ≥ 50%, no difference in cardiac death was observed between two groups (*p* = .990).

### 3.4 Per‐protocol analysis (successful CTO PCI vs. MT/failed PCI)

**Figure 3:**
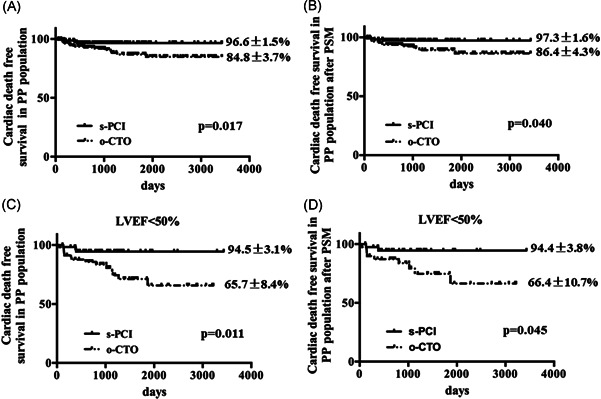
Kaplan–Meier analysis of primary endpoints in PP population. (A) in overall population. (B) after PSM. (C) in patients with LVEF < 50%. (D) in patients with LVEF < 50% after PSM. LVEF, left ventricular ejection fraction; o‐CTO, occluded chronic total occlusion; PP, per‐protocol; PSM, propensity score matching; s‐PCI, successful percutaneous coronary intervention

As shown in Figure 1, technical success was achieved in 166 patients, who were classified into the successful PCI (s‐PCI) group (*n* = 166). Thirty‐two patients failed in the PCI procedure and 132 patients receiving MT constituted the occluded CTO (o‐CTO) group (*n* = 164). The baseline characteristics in per‐protocol analysis before and after PSM are shown in Table S1. The results of per‐protocol analysis showed a significantly higher incidence of cardiac death‐free survival in the s‐PCI group both before (96.6% vs. 84.8%, *p* = .017) and after PSM (97.3% vs. 86.4%, *p* = .040) when compared with those in the o‐CTO group (Table S2 and Figure 3A,B).

In subgroup analysis in the per‐protocol population, the long‐term cardiac death‐free survival is higher in the s‐PCI group compared with the o‐CTO group in patients with LVEF < 50% before and after PSM (Figure 3C,D). Similarly, no significant difference was noted between the two groups in the occurrence of cardiac death in patients with LVEF ≥ 50% (*p* = .70).

### 3.5 Independent predictors of cardiac death

By univariate analysis, age (hazard ratio [HR]: 1.08, 95% confidence interval [CI]: 1.04–1.21, *p* < .001), LVEF < 50% (HR: 5.87, 95% CI: 2.16–15.91, *p* < .001), and LAD IRA (HR: 2.59, 95% CI: 1.06–6.36, *p* = .038) were positively associated with long‐term cardiac death, while successful CTO PCI (HR: 0.31, 95% CI: 0.12–0.85, *p* = .023) was negatively associated with cardiac death. By multivariate analysis, age (HR: 1.06, 95% CI: 1.02–1.10, *p* = .003) and LVEF < 50% (HR: 4.71, 95% CI: 1.7–12.90, *p* = .003) remained significantly correlated with long‐term cardiac death; however, successful CTO PCI showed borderline significance (HR: 0.42, 95% CI: 0.15–1.16, *p* = .095) and LAD IRA become insignificant (Table S3).

### 3.6 Successful versus failed CTO PCI

The procedural characteristics and in‐hospital results of CTO PCI are listed in Table S4. In this study, most patients (86.9%) underwent CTO PCI within three months after AMI. More contrast volume (273.5 ± 119.2 ml vs. 205.0 ± 107.0 ml, *p* = .003) and longer procedural time (109.4 ± 61.4 min vs. 79.2 ± 42.4 min, *p* = .015) was observed in successful compared with failed CTO PCI group. There was no significant difference in in‐hospital complications between the two groups.

## 4 DISCUSSION

The main findings of the current study were as follows. (1) PCI of CTO in non‐IRA was associated with a higher cardiac death‐free survival during long‐term follow‐up compared with MT. (2) PCI was beneficial in patients over 65 years old, with LVEF < 50%, LAD IRA, and non‐LAD CTO lesion after IRA PCI. (3) Age and LVEF < 50% were independent predictors of long‐term cardiac death, while successful CTO PCI only showed borderline significance. (4) Patients with LVEF < 50% who underwent successful PCI in non‐IRA CTO lesion had a significantly lower risk of cardiac death than those with occluded CTO lesion. (5) In‐hospital complications were not different between successful and failed CTO PCI groups.

The adequacy of revascularization of CTO remains controversial in consideration of the procedural complexity, low success rate, and frequent complications. In this study, the success rate of CTO‐PCI is 83.8% (166/198). The in‐hospital complication rate is comparable between successful (1.2%) and failed (6.2%) CTO PCI groups. Although observational studies and meta‐analysis have reported better clinical outcomes for CTO‐PCI in patients with stable coronary artery disease (CAD),^19^, ^20^ none of the randomized clinical trials showed a difference in MACE at long‐term follow‐up for CTO‐PCI compared with optimal medical therapy, which makes the treatment strategy for CTO still controversial.^21^, ^22^, ^23^, ^24^

In patients with AMI, the presence of concurrent CTO is associated with increased mortality.^3^, ^6^ The pathological mechanisms involved include aggravated ischemia caused by occlusion of IRA and subsequent interruption of collateral supply to non‐IRA CTO, microvascular ischemia, reperfusion injury, and electrical instability, which may lead to poorer outcomes in these patients compared with patients who suffered from CTO and stable CAD.^25^ Theoretically, revascularization of non‐IRA CTO after IRA PCI might yield more clinical benefits, as the recovery of blood supply in both the CTO territory and overlapping border of the infarct zone may reduce left ventricular remodeling and improve contractile function.

Based on this theory, several observational studies have been performed in patients with AMI (including STEMI and NSTEMI) and concurrent non‐IRA CTO. Park et al.,^10^ identified 422 patients from the Korean CTO registry and showed a lower incidence of MACE (12.5% vs. 17.8%) and cardiac death (4.0% vs. 9.9%) in the successful CTO‐PCI group compared with failed CTO‐PCI group at one‐year follow‐up. Similarly, Choi et al.,^9^ enrolled 324 patients from COREA‐AMI Registry and demonstrated a reduced prevalence of MACE (21.9% vs. 55.2%) and cardiac death (7.6% vs. 20.1%) in successful CTO‐PCI group compared with occluded CTO group at 5‐year follow up. In our study, the analysis based on the per‐protocol population demonstrated that patients who underwent successful CTO PCI exhibited lower rates of cardiac death compared to patients with occluded CTO lesion both before and after PSM. Of note, successful CTO PCI showed borderline significance (0.095) as a predictor of cardiac death in multivariate analysis. Therefore, we cautiously suggest that s‐PCI in non‐IRA CTO has a beneficial effect after AMI. Although similar clinical benefits were demonstrated in the PCI group according to ITT analysis, no significant difference was found in the rate of cardiac death between PCI and MT groups after PSM. As failed CTO‐PCI is associated with a higher incidence of MACE compared with successful CTO‐PCI,^26^ the ITT analysis may lead to underestimation of the actual effect of successful CTO‐PCI. Also, patients in failed CTO‐PCI group usually had higher baseline clinical risks,^27^ which contributed to poor prognosis in these patients. Therefore, a prospective, randomized trial would be necessary to investigate the beneficial effect of successful CTO PCI.

The subgroup analysis from the Korean CTO registry, COREA‐AMI Registry, and a retrospective study from Teng et al.,^9^ demonstrated a lower incidence of all‐cause mortality in NSTEMI patients undergoing successful CTO‐PCI during 1‐ to 5‐year follow‐up.^9^, ^10^, ^28^ However, in the NSTEMI subgroup of this study, the prevalence of cardiac death was not significantly different between the s‐PCI and the o‐CTO groups (Figure S2). Potential explanations for the different results in these studies might be the non‐randomized design and small sample size, which warrant further randomized trials in this subgroup of patients.

Previous observational studies indicated that STEMI patients with non‐IRA CTO also benefit from CTO‐PCI. The 1‐year clinical outcomes demonstrated reduced MACE (19.5% vs. 34.6%) and cardiac death (1.7%–3.6% vs. 12.0%–15.4%) in CTO‐PCI compared with MT (including failed or non‐attempted CTO).^11^, ^29^ During the long‐term follow‐up (mean period between 4 and 6 years), patients treated with staged CTO‐PCI still showed lower MACE (18.9%–22.0% vs. 46.9%–48.4%) and cardiac death (4.0%–4.4% vs. 15.0%–16.8%).^11^, ^12^, ^30^ However, except for relieving angina in the CTO‐PCI group (94% vs. 87%, *p* = .03), the only randomized trial EXPLORE failed to find any difference in MACE (7.4% vs. 6.5%) and cardiac death (2.7% vs. 0%) between CTO‐PCI and CTO‐No PCI group at 1‐year follow‐up. Moreover, at 4‐year follow‐up, cardiac death was significantly higher in the CTO‐PCI group (6.0% vs. 1.0%, *p* = .02), while no difference in MACE was observed.^31^ The high cardiac death at the CTO‐PCI group in this study may be explained by the short time interval (5 ± 2 days) between IRA PCI and CTO‐PCI, during which inflammation plays an important role and lead to larger infarct size and adverse left ventricular remodeling. In the current study, almost half of patients completed CTO‐PCI within 1 week and most patients completed CTO‐PCI within 3 months after IRA PCI. By univariate analysis, CTO‐PCI within 1 week is not identified as an independent predictor of cardiac death. Thus, the appropriate timing of staged CTO‐PCI after IRA PCI for AMI is still unknown.

Recently, Ito et al.^32^ demonstrated that non‐IRA CTO was an independent prognostic factor for all‐cause death and MACE only in the reduced EF group (LVEF < 50%), but not in the preserved EF group(LVEF ≥ 50%). Similarly, we identified LVEF < 50% as an independent predictor of long‐term cardiac death in patients with AMI and concurrent CTO, which is consistent with a retrospective study by Yoshida et al.,^30^ implying a close relationship between CTO with reduced LVEF and worse clinical outcomes. The subgroup analysis of the current study showed that successful CTO‐PCI is associated with better clinical outcomes in patients with LVEF < 50%, which is consistent with recently published data showing that only patients with reduced EF benefit from successful staged CTO‐PCI after AMI.^32^ However, this group of patients usually has high cardiovascular risks and are excluded from randomized trials related to CTO‐PCI. This may partially explain why none of the conducted randomized studies showed significant benefit of CTO‐PCI in clinical outcomes compared with MT.

In contrast with previous reports showing improved LVEF and long‐term survival in PCI for LAD CTO lesion,^15^, ^33^ the subgroup analysis in our study demonstrated that PCI was associated with less cardiac death in non‐LAD CTO lesion. Recently, Choi et al.^34^ also reported in a retrospective study that the 5‐year cumulative incidence of the composite of total death or myocardial infarction was significantly lower in patients who underwent non‐LAD CTO PCI than patients receiving MT. To our knowledge, myocardial viability is associated with long‐term outcomes after CTO PCI,^35^ and the prognosis of CTO‐PCI may differ due to the amount of myocardium at risk, which is supplied by the CTO vessel. As myocardial viability data was not available in retrospective studies, the benefit of CTO revascularization in a single vessel would be difficult to determine.

## 5 CONCLUSION

In conclusion, patients undergoing successful revascularization of non‐IRA CTO after AMI might have a better long‐term prognosis compared with patients with o‐CTO. LVEF < 50% is an independent predictor of cardiac death and patients with LVEF < 50% may benefit from successful CTO‐PCI after AMI.

## 6 LIMITATIONS

There are several limitations to this study. First, this is a retrospective observational study in a single center. Therefore, a limited number of patients were included. Second, the selection of CTO‐PCI or not and the timing of CTO‐PCI after IRA PCI is left to the preferences of patients and doctors. Therefore, the potential for selection bias cannot be excluded. Third, the myocardial viability test was not routinely performed, and potential imbalances of the amount of viable myocardium may influence the clinical outcomes.

## CONFLICT OF INTERESTS

The authors declare that there are no conflict of interests.

## Supporting information

Supporting information.

Supporting information.

Supporting information.

Supporting information.

Supporting information.

Supporting information.

Supporting information.

## Data Availability

The datasets generated during and/or analyzed during the current study are available from the corresponding author on reasonable request.

## References

[1] Park HW , Yoon CH , Kang SH , et al. Early‐ and late‐term clinical outcome and their predictors in patients with ST‐segment elevation myocardial infarction and non‐ST‐segment elevation myocardial infarction. Int J Cardiol. 2013;169(4):254‐261. 24071385 10.1016/j.ijcard.2013.08.132

[2] Jensen LO , Terkelsen CJ , Horváth‐Puhó E , et al. Influence of multivessel disease with or without additional revascularization on mortality in patients with ST‐segment elevation myocardial infarction. Am Heart J. 2015;170(1):70‐78. 26093866 10.1016/j.ahj.2015.03.020

[3] Claessen BE , Dangas GD , Weisz G , et al. Prognostic impact of a chronic total occlusion in a non‐infarct‐related artery in patients with ST‐segment elevation myocardial infarction: 3‐year results from the HORIZONS‐AMI trial. Eur Heart J. 2012;33(6):768‐775. 22240495 10.1093/eurheartj/ehr471

[4] Hoebers LP , Vis MM , Claessen BE , et al. The impact of multivessel disease with and without a co‐existing chronic total occlusion on short‐ and long‐term mortality in ST‐elevation myocardial infarction patients with and without cardiogenic shock. Eur J Heart Fail. 2013;15(4):425‐432. 23148116 10.1093/eurjhf/hfs182

[5] Stähli BE , Varbella F , Schwarz B , et al. Rationale and design of the MULTISTARS AMI Trial: a randomized comparison of immediate versus staged complete revascularization in patients with ST‐segment elevation myocardial infarction and multivessel disease. Am Heart J. 2020;228:98‐108. 32871329 10.1016/j.ahj.2020.07.016

[6] Watanabe H , Morimoto T , Shiomi H , et al. Chronic total occlusion in a non‐infarct‐related artery is closely associated with increased five‐year mortality in patients with ST‐segment elevation acute myocardial infarction undergoing primary percutaneous coronary intervention (from the CREDO‐Kyoto AMI registry). EuroIntervention. 2017;12(15):e1874‐e1882. 28044983 10.4244/EIJ-D-15-00421

[7] Claessen BE , van der Schaaf RJ , Verouden NJ , et al. Evaluation of the effect of a concurrent chronic total occlusion on long‐term mortality and left ventricular function in patients after primary percutaneous coronary intervention. JACC Cardiovasc Interv. 2009;2(11):1128‐1134. 19926056 10.1016/j.jcin.2009.08.024

[8] An X , Yang J , Dou K , Yang Y . The 11‐year prognostic impact of chronic total occlusion in the noninfarct‐related coronary artery on patients with acute myocardial infarction. J Interv Cardiol. 2021;2021:6646804. 33824627 10.1155/2021/6646804 PMC7994075

[9] Choi IJ , Koh YS , Lim S , et al. Impact of percutaneous coronary intervention for chronic total occlusion in non‐infarct‐related arteries in patients with acute myocardial infarction (from the COREA‐AMI registry). Am J Cardiol. 2016;117(7):1039‐1046. 26993974 10.1016/j.amjcard.2015.12.049

[10] Park JY , Choi BG , Rha SW , et al. Chronic total occlusion intervention of the non‐infarct‐related artery in acute myocardial infarction patients: the Korean multicenter chronic total occlusion registry. Coron Artery Dis. 2018;29(6):495‐501. 29688904 10.1097/MCA.0000000000000630

[11] Valenti R , Marrani M , Cantini G , et al. Impact of chronic total occlusion revascularization in patients with acute myocardial infarction treated by primary percutaneous coronary intervention. Am J Cardiol. 2014;114(12):1794‐1800. 25438904 10.1016/j.amjcard.2014.09.016

[12] Cui KY , Yuan F , Liu H , et al. Long‐term outcomes of staged recanalization for concurrent chronic total occlusion in patients with ST‐segment elevation myocardial infarction after primary percutaneous coronary intervention. J Geriatr Cardiol. 2020;17(1):16‐25. 32133033 10.11909/j.issn.1671-5411.2020.01.010 PMC7008095

[13] Villablanca PA , Olmedo W , Weinreich M , et al. Staged percutaneous intervention for concurrent chronic total occlusions in patients with st‐segment‐elevation myocardial infarction: a systematic review and meta‐analysis. J Am Heart Assoc. 2018;7(8):e008415. 29654206 10.1161/JAHA.117.008415 PMC6015413

[14] Tong J , Yu Q , Li C , Shao X , Xia Y . Successful revascularization of noninfarct related artery with chronic total occlusion among acute myocardial infarction patients: a systematic review and meta‐analysis. Medicine. 2018;97(3):e9655. 29505003 10.1097/MD.0000000000009655 PMC5779772

[15] Henriques JP , Hoebers LP , Råmunddal T , et al. Percutaneous intervention for concurrent chronic total occlusions in patients with STEMI: the EXPLORE Trial. J Am Coll Cardiol. 2016;68(15):1622‐1632. 27712774 10.1016/j.jacc.2016.07.744

[16] Thygesen K , Alpert JS , Jaffe AS , et al. Fourth universal definition of myocardial infarction (2018). Circulation. 2018;138(20):e618‐e651. 30571511 10.1161/CIR.0000000000000617

[17] Ybarra LF , Rinfret S , Brilakis ES , et al. Definitions and clinical trial design principles for coronary artery chronic total occlusion therapies: CTO‐ARC consensus recommendations. Circulation. 2021;143(5):479‐500. 33523728 10.1161/CIRCULATIONAHA.120.046754

[18] Schulte PJ , Mascha EJ . Propensity score methods: theory and practice for anesthesia research. Anesth Analg. 2018;127(4):1074‐1084. 29750691 10.1213/ANE.0000000000002920

[19] Li KHC , Wong KHG , Gong M , et al. Percutaneous coronary intervention versus medical therapy for chronic total occlusion of coronary arteries: a systematic review and meta‐analysis. Curr Atheroscler Rep. 2019;21(10):42. 31399762 10.1007/s11883-019-0804-8 PMC6689032

[20] Megaly M , Saad M , Tajti P , et al. Meta‐analysis of the impact of successful chronic total occlusion percutaneous coronary intervention on left ventricular systolic function and reverse remodeling. J Interv Cardiol. 2018;31(5):562‐571. 29974508 10.1111/joic.12538

[21] Werner GS , Martin‐Yuste V , Hildick‐Smith D , et al. A randomized multicentre trial to compare revascularization with optimal medical therapy for the treatment of chronic total coronary occlusions. Eur Heart J. 2018;39(26):2484‐2493. 29722796 10.1093/eurheartj/ehy220

[22] Obedinskiy AA , Kretov EI , Boukhris M , et al. The IMPACTOR‐CTO Trial. JACC Cardiovasc Interv. 2018;11(13):1309‐1311. 29976368 10.1016/j.jcin.2018.04.017

[23] Mashayekhi K , Nührenberg TG , Toma A , et al. A randomized trial to assess regional left ventricular function after stent implantation in chronic total occlusion: the REVASC Trial. JACC Cardiovasc Interv. 2018;11(19):1982‐1991. 30219327 10.1016/j.jcin.2018.05.041

[24] Lee SW , Lee PH , Ahn JM , et al. Randomized trial evaluating percutaneous coronary intervention for the treatment of chronic total occlusion. Circulation. 2019;139(14):1674‐1683. 30813758 10.1161/CIRCULATIONAHA.118.031313

[25] Kim SH , Behnes M , Mashayekhi K , et al. Prognostic impact of percutaneous coronary intervention of chronic total occlusion in acute and periprocedural myocardial infarction. J Clin Med. 2021;10(2). 10.3390/jcm10020258 PMC7828144 33445664

[26] Megaly M , Khalil M , Basir MB , et al. Outcomes of successful vs. failed contemporary chronic total occlusion percutaneous coronary intervention. Cardiovasc Interv Ther. 2021 10.1007/s12928-021-00819-x 34716883

[27] Christakopoulos GE , Christopoulos G , Carlino M , et al. Meta‐analysis of clinical outcomes of patients who underwent percutaneous coronary interventions for chronic total occlusions. Am J Cardiol. 2015;115(10):1367‐1375. 25784515 10.1016/j.amjcard.2015.02.038

[28] Teng HI , Sung SH , Huang SS , et al. The impact of successful revascularization of coronary chronic total occlusions on long‐term clinical outcomes in patients with non‐ST‐segment elevation myocardial infarction. J Interv Cardiol. 2018;31(3):302‐309. 29495125 10.1111/joic.12501

[29] Deng J , Wang X , Shi Y , Zhao X , Han Y . Prognostic value of the age, creatinine, and ejection fraction score for non‐infarct‐related chronic total occlusion revascularization after primary percutaneous intervention in acute ST‐elevation myocardial infarction patients: a retrospective study. J Interv Cardiol. 2018;31(1):33‐40. 28940388 10.1111/joic.12448

[30] Yoshida R , Ishii H , Morishima I , et al. Prognostic impact of recanalizing chronic total occlusion in non‐infarct related arteries on long‐term clinical outcomes in acute myocardial infarction patients undergoing primary percutaneous coronary intervention. Cardiovasc Interv Ther. 2020;35(3):259‐268. 31456091 10.1007/s12928-019-00615-8

[31] Elias J , van Dongen IM , Råmunddal T , et al. Long‐term impact of chronic total occlusion recanalisation in patients with ST‐elevation myocardial infarction. Heart. 2018;104(17):1432‐1438. 29463612 10.1136/heartjnl-2017-312698

[32] Ito H , Masuda J , Kurita T , et al. Effect of left ventricular ejection fraction on the prognostic impact of chronic total occlusion in a non‐infarct‐related artery in patients with acute myocardial infarction. Int J Cardiol Heart Vasc. 2021;33:100738. 33718588 10.1016/j.ijcha.2021.100738 PMC7933260

[33] Safley DM , House JA , Marso SP , Grantham JA , Rutherford BD . Improvement in survival following successful percutaneous coronary intervention of coronary chronic total occlusions: variability by target vessel. JACC Cardiovascular interventions. 2008;1(3):295‐302. 19463316 10.1016/j.jcin.2008.05.004

[34] Choi JY , Rha SW , Choi BG , et al. Percutaneous coronary intervention for chronic total occlusion in single coronary arteries. Tex Heart Inst J. 2021;48(2). 10.14503/THIJ-19-7023 PMC8262825 34111277

[35] Song L , Qiao S , Guan C , et al. Association of symptom status, myocardial viability, and clinical/anatomic risk on long‐term outcomes after chronic total occlusion percutaneous coronary intervention. Catheter Cardiovasc Interv. 2021;97(Suppl 2):996‐1008. 33650804 10.1002/ccd.29577

