# Supplementary figures and images for: A comparison of long‐term clinical outcomes between percutaneous coronary intervention (PCI) and medical therapy in patients with chronic total occlusion in noninfarct‐related artery after PCI of acute myocardial infarction
Source: Clin Cardiol. 2022 Jan 6;45(1):136–44. doi: 10.1002/clc.23771 (PMC8799053; doi:10.1002/clc.23771)

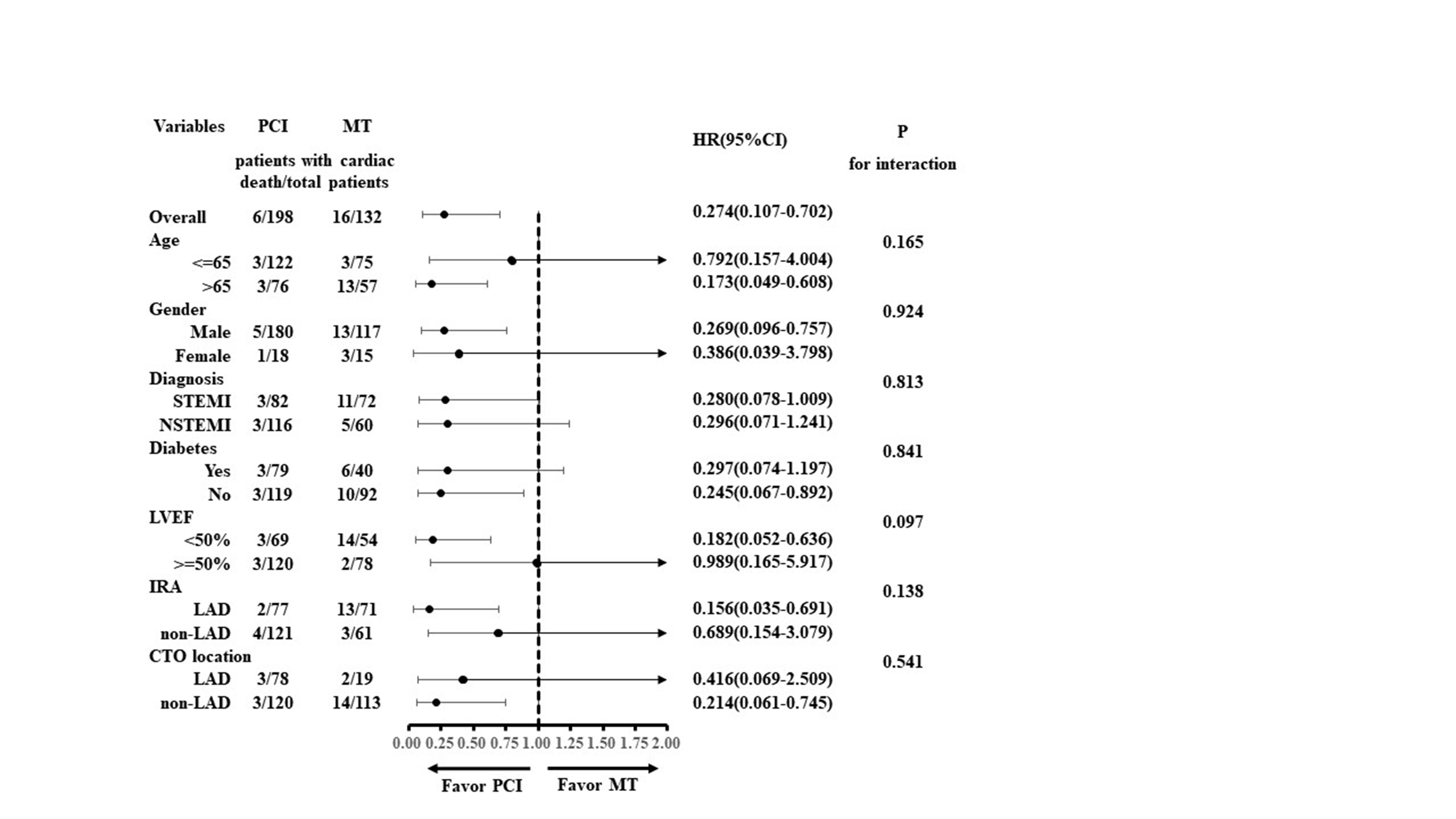

Supplement: Supplementary file 1 — Supporting information. [file CLC-45-136-s007.jpg]

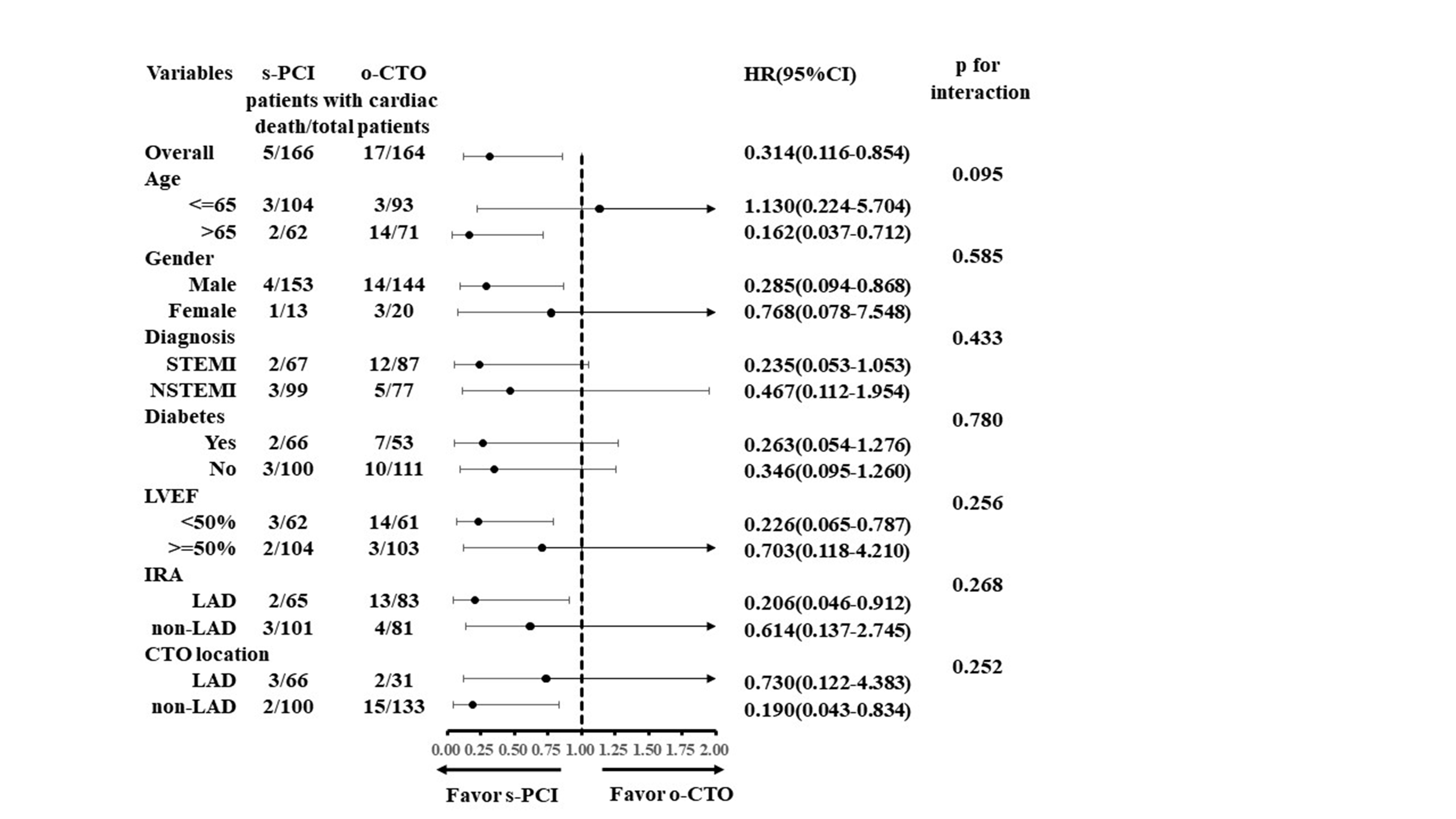

Supplement: Supplementary file 2 — Supporting information. [file CLC-45-136-s003.jpg]
