# Supplementary material for: A comparison of long‐term clinical outcomes between percutaneous coronary intervention (PCI) and medical therapy in patients with chronic total occlusion in noninfarct‐related artery after PCI of acute myocardial infarction
Source: Clin Cardiol. 2022 Jan 6;45(1):136–44. doi: 10.1002/clc.23771 (PMC8799053; doi:10.1002/clc.23771)
Supplement: Supplementary file 3 — Supporting information. [file CLC-45-136-s001.docx]

| **Supplementary Table 1 Baseline and procedural characteristics during AMI PCI in per-protocol population** | | | | | | | | | |  |
| --- | --- | --- | --- | --- | --- | --- | --- | --- | --- | --- |
|  | All patients | |  | Standardized | Matched patients | |  | Standardized | |  |
|  | s-PCI(n=166) | o-CTO(n=164) | p value | difference | s-PCI(n=119) | o-CTO(n=119) | p value | difference | |  |
| Male(%) | 153(92.2) | 144(87.8) | 0.186 | 0.147 | 109(91.6) | 108(90.8) | 0.819 | 0.028 | |  |
| Age(years) | 60.4±11.5 | 64.2±12.4 | 0.005 | -0.318 | 61.6±11.6 | 62.0±12.8 | 0.807 | -0.033 | |  |
| Hypertension(%) | 112(67.5) | 109(66.5) | 0.846 | 0.021 | 79(66.4) | 81(68.1) | 0.782 | -0.036 | |  |
| Diabetes(%) | 66(39.8) | 53(32.3) | 0.159 | 0.157 | 47(39.5) | 46(38.7) | 0.894 | 0.016 | |  |
| Insulin(%) | 10(6.0) | 16(9.8) | 0.208 | -0.141 | 6(5.0) | 14(11.8) | 0.100 | -0.247 | |  |
| Dyslipidemia(%) | 14(8.4) | 13(7.9) | 0.867 | 0.018 | 13(10.9) | 8(6.7) | 0.253 | 0.149 | |  |
| Current smoking(%) | 56(33.7) | 62(37.8) | 0.441 | -0.086 | 40(33.6) | 45(37.8) | 0.499 | -0.088 | |  |
| Previous MI(%) | 37(22.3) | 31(18.9) | 0.447 | 0.084 | 24(20.2) | 27(22.7) | 0.636 | -0.060 | |  |
| Previous PCI (%) | 32(19.3) | 31(18.9) | 0.931 | 0.010 | 22(18.5) | 25(21.0) | 0.625 | -0.063 | |  |
| Diagnosis |  |  |  |  |  |  |  |  | |  |
| STEMI（%） | 67(40.4) | 87(53.0) | 0.021 | -0.255 | 50(42.0) | 56(47.1) | 0.434 | -0.103 | |  |
| Lab test |  |  |  |  |  |  |  |  | |  |
| TC(mmol/L) | 4.4±1.2 | 4.4±1.1 | 0.792 | <0.001 | 4.5±1.2 | 4.4±1.1 | 0.384 | 0.087 | |  |
| TG(mmol/L) | 2.0±1.4 | 1.8±1.3 | 0.227 | 0.148 | 2.0±1.4 | 2.0±1.4 | 0.751 | <0.001 | |  |
| LDL-C(mmol/L) | 2.6±1.1 | 2.6±1.1 | 0.523 | <0.001 | 2.7±1.1 | 2.6±1.1 | 0.476 | 0.091 | |  |
| HDL-C(mmol/L) | 1.0±0.3 | 1.0±0.3 | 0.445 | <0.001 | 1.0±0.3 | 1.0±0.3 | 0.580 | <0.001 | |  |
| eGFR | 86.8±24.2 | 78.6±29.1 | 0.006 | 0.306 | 86.4±26.1 | 80.8±29.8 | 0.129 | 0.200 | |  |
| (mL/min/1.73 m2) |  |  |  |  |  |  |  |  | |  |
| HbA1c(%) | 6.8±1.7 | 6.8±1.7 | 0.916 | <0.001 | 6.7±1.7 | 6.9±1.8 | 0.468 | -0.114 | |  |
| LVEF(%) | 52.7±10.1 | 52.1±10.5 | 0.559 | 0.058 | 52.8±9.8 | 52.2±10.5 | 0.651 | 0.059 | |  |
| LVEF<50% | 62(37.3) | 61(37.2) | 0.977 | 0.002 | 41(34.5) | 42(35.3) | 0.892 | -0.017 | |  |
| Infarct-related artery |  |  |  |  |  |  |  |  | |  |
| LM(%) | 1(0.6) | 0 | >0.999 | - | 0 | 0 | - | - | |  |
| LAD(%) | 64(38.6) | 83(50.6) | 0.028 | -0.243 | 57(47.9) | 61(51.3) | 0.604 | -0.068 | |  |
| LCX(%) | 42(25.3) | 29(17.7) | 0.092 | 0.186 | 25(21.0) | 23(19.3) | 0.747 | 0.042 | |  |
| RCA(%) | 60(36.1) | 56(34.1) | 0.704 | 0.042 | 37(31.1) | 35(29.4) | 0.778 | 0.037 | |  |
| In stent thrombosis(%) | 4(2.4) | 7(4.3) | 0.377 | -0.106 | 3(2.5) | 5(4.2) | 0.722 | -0.095 | |  |
| Location of CTO |  |  |  |  |  |  |  |  | |  |
| LAD(%) | 66(39.8) | 31(18.9) | <0.001 | 0.472 | 31(26.1) | 27(22.7) | 0.546 | 0.079 | |  |
| LCX(%) | 45(27.1) | 74(45.1) | 0.001 | -0.382 | 44(37.0) | 47(39.5) | 0.689 | -0.051 | |  |
| RCA(%) | 61(36.7) | 65(39.6) | 0.589 | -0.069 | 48(40.3) | 49(41.2) | 0.895 | -0.018 | |  |
| In stent CTO(%) | 7(4.2) | 8(4.9) | 0.798 | -0.034 | 6(5.0) | 6(5.0) | >0.999 | <0.001 | |  |
| IABP use (%) | 2(1.2) | 2(1.2) | >0.999 | <0.001 | 2(1.7) | 1(0.8) | >0.999 | 0.081 | |  |
| Peak troponin T (ng/mL) | 0.58 | 1.17 | 0.033 | -0.232 | 0.69 | 0.99 | 0.473 | -0.058 | |  |
|  | (0.18-2.18) | (0.24-4.39) |  |  | (0.21-2.81) | (0.23-3.5) |  |  | |  |
| Peak CK-MB(U/L) | 23(16-61) | 34(16-122) | 0.146 | -0.168 | 25(16-70) | 25(15-65) | 0.755 | 0.006 | |  |
| Stents/patient | 1.6±0.7 | 1.5±0.7 | 0.175 | 0.143 | 1.6±0.7 | 1.4±0.7 | 0.083 | 0.286 | |  |
| Average stent diameter | 3.1±0.4 | 3.0±0.4 | 0.376 | 0.250 | 3.1±0.4 | 3.0±0.4 | 0.739 | 0.250 | |  |
| (mm) |  |  |  |  |  |  |  |  | |  |
| Total stent length(mm) | 46.5±25.3 | 43.9±22.8 | 0.325 | 0.108 | 46.1±26.3 | 43.2±20.4 | 0.345 | 0.123 | |  |
| Abbreviations: s-PCI:successful percutaneous coronary intervention; MT: medical therapy; PSM: propensity score matching; MI: myocardial infarction; STEMI:ST-segment elevation myocardial infarction; NSTEMI:non-ST segment elevation myocardial infarction; TC: total cholesterol; TG:triglyceride; LDL-C: low-density lipoprotein cholesterol; HDL-C: high-density lipoprotein cholesterol; eGFR: estimated glomerular filtration rate; HbA1c: hemoglobin A1c; LVEF: left ventricular ejection fraction; LM: left main coronary artery; LAD: left anterior descending coronary artery; LCX: left circumflex coronary artery; RCA: right coronary artery; CTO: chronic total occlusion; IABP: intra-aortic balloon pump | | | | | | | | |  |  |
|  |  |  |  |  |  |  |  |  |  | |
|  |  |  |  |  |  |  |  |  |  | |
|  |  |  |  |  |  |  |  |  |  | |
|  |  |  |  |  |  |  |  |  |  | |
|  |  |  |  |  |  |  |  |  |  | |
|  |  |  |  |  |  |  |  |  |  | |
