# Supplementary material for: A comparison of long‐term clinical outcomes between percutaneous coronary intervention (PCI) and medical therapy in patients with chronic total occlusion in noninfarct‐related artery after PCI of acute myocardial infarction
Source: Clin Cardiol. 2022 Jan 6;45(1):136–44. doi: 10.1002/clc.23771 (PMC8799053; doi:10.1002/clc.23771)
Supplement: Supplementary file 4 — Supporting information. [file CLC-45-136-s006.docx]

| **Supplementary Table 2 Long term clinical outcomes in per-protocol population** | | | | | | |  |
| --- | --- | --- | --- | --- | --- | --- | --- |
|  | All patients | |  | PSM patients | |  |  |
|  | s-PCI (n=166) | o-CTO (n=164) | p value | s-PCI (n=119) | o-CTO (n=119) | p value |  |
|  |  |  |  |  |  |  |  |
| All cause death(%) | 9(5.4) | 23(14.0) | 0.030 | 6(5.0) | 15(12.6) | 0.080 |  |
| Cardiac death(%) | 5(3.0) | 17(10.4) | 0.017 | 3(2.5) | 11(9.2) | 0.040 |  |
| MI(%) | 8(4.8) | 12(7.3) | 0.660 | 8(6.7) | 9(7.6) | 0.840 |  |
| Stroke(%) | 2(1.2) | 8(4.9) | 0.120 | 2(1.7) | 5(4.2) | 0.310 |  |
| Revascularization(%) | 28(16.9) | 26(15.9) | 0.360 | 22(18.5) | 20(16.8) | 0.360 |  |
| CTO vessel(%) | 14(8.4) | 9(5.5) | - | 11(9.2) | 6(5.0) | - |  |
| Infarct-related artery(%) | 8(4.8) | 11(6.7) | - | 7(5.9) | 9(7.6) | - |  |
| Other(%) | 12(7.2) | 13(7.9) | - | 9(7.6) | 11(9.2) | - |  |
| MACCE(%) | 38(22.9) | 55(33.5) | 0.290 | 29(24.4) | 39(32.8) | 0.560 |  |
| Abbreviations:s-PCI:successful percutaneous coronary intervention; o-CTO: occluded chronic total occlusion; PSM: propensity score matching; MI: myocardial infarction; MACCE: major adverse cardiovascular and cerebrovascular events. | | | | | | |  |
|  |  |  |  |  |  |  |  |
|  |  |  |  |  |  |  |  |
