# Supplementary material for: A comparison of long‐term clinical outcomes between percutaneous coronary intervention (PCI) and medical therapy in patients with chronic total occlusion in noninfarct‐related artery after PCI of acute myocardial infarction
Source: Clin Cardiol. 2022 Jan 6;45(1):136–44. doi: 10.1002/clc.23771 (PMC8799053; doi:10.1002/clc.23771)
Supplement: Supplementary file 5 — Supporting information. [file CLC-45-136-s004.docx]

| **Supplementary Table 3 Univariate and multivariate adjusted predictors of cardiac death** | | | |  |  |
| --- | --- | --- | --- | --- | --- |
| Variable | Unadjusted HR | P value | Multivariate adjusted HR | P value |  |
|  | (95% Confidence Interval) |  | (95% Confidence Interval) |  |  |
| Age | 1.08(1.04-1.12) | <0.001 | 1.06(1.02-1.10) | 0.003 |  |
| Male gender | 0.50(0.17-1.49) | 0.216 |  |  |  |
| Diabetes | 1.21(0.52-2.82) | 0.666 |  |  |  |
| Previous PCI | 1.02(0.35-3.02) | 0.972 |  |  |  |
| Previous MI | 0.64(0.19-2.16) | 0.468 |  |  |  |
| STEMI | 1.98(0.83-4.73) | 0.124 |  |  |  |
| eGFR | 0.99(0.97-1.00) | 0.097 |  |  |  |
| peak cTnT | 1.09(0.98-1.21) | 0.133 |  |  |  |
| LVEF<50% | 5.87(2.16-15.91) | <0.001 | 4.71(1.72-12.90) | 0.003 |  |
| LAD IRA | 2.59(1.06-6.36) | 0.038 | 2.13(0.86-5.26) | 0.100 |  |
| LAD CTO | 0.78(0.29-2.13) | 0.631 |  |  |  |
| LCX CTO | 1.55(0.67-3.59) | 0.304 |  |  |  |
| Successful CTO PCI | 0.31(0.12-0.85) | 0.023 | 0.42(0.15-1.16) | 0.095 |  |
| Abbreviations: HR: hazard ratio; PCI:percutaneous coronary intervention; MI: myocardial infarction; STEMI:ST-segment elevation myocardial infarction; eGFR: estimated glomerular filtration rate; LVEF: left ventricular ejection fraction; LAD: left anterior descending coronary artery; LCX: left circumflex coronary artery; CTO: chronic total occlusion. | | | | |  |
|  |  |  |  |  |  |
|  |  |  |  |  |  |
