# Supplementary material for: A comparison of long‐term clinical outcomes between percutaneous coronary intervention (PCI) and medical therapy in patients with chronic total occlusion in noninfarct‐related artery after PCI of acute myocardial infarction
Source: Clin Cardiol. 2022 Jan 6;45(1):136–44. doi: 10.1002/clc.23771 (PMC8799053; doi:10.1002/clc.23771)
Supplement: Supplementary file 6 — Supporting information. [file CLC-45-136-s005.docx]

| **Supplementary Table4 Procedural characteristics and clinical outcomes in CTO PCI** | | | |  |
| --- | --- | --- | --- | --- |
|  | CTO PCI | |  |  |
|  | successful(n=166) | failed(n=32) | p value |  |
| **Procedural characteristics** | | | |  |
| Multiple CTO attemps(%) | 13(7.8) | 4(12.5) | 0.388 |  |
| Multiple CTO lesions(%) | 2(1.2) | 2(6.3) | 0.123 |  |
| CTO PCI interval after primary PCI | |  | 0.437 |  |
| Within one week | 75(45.2) | 16(50.0) |  |  |
| One week to one month | 12(7.2) | 3(9.3) |  |  |
| One to three month | 59(35.5) | 7(21.8) |  |  |
| Three month to one year | 20(12.0) | 6(18.5) |  |  |
| CTO technique |  |  | 0.539 |  |
| antegrade only | 138(83.1) | 28(87.5) |  |  |
| antegrade+retrograde | 28(16.9) | 4(12.5) |  |  |
| Number of CTO wires used | 2.0±1.3 | 1.9±1.2 | 0.813 |  |
| IVUS use (%) | 27(16.3) | 2(6.3) | 0.142 |  |
| Stents/patient | 2.0±1.0 | - |  |  |
| Average stent diameter（mm） | 2.9±0.3 | - |  |  |
| Total stent length（mm） | 65.4±28.6 | - |  |  |
| Contrast volume（mL） | 273.5±119.2 | 205.0±107.0 | 0.003 |  |
| Procedural time（min） | 109.4±61.4 | 79.2±42.4 | 0.015 |  |
| **In-hospital complications** |  |  |  |  |
| Peri-procedural MI | 0 | 1(3.1) | - |  |
| Perforation | 2(1.2) | 0 | - |  |
| Cardiac tamponade | 0 | 0 | - |  |
| Ischemia-driven TVR | 0 | 1(3.1) | - |  |
| Abbreviations: CTO: chronic total occlusion; PCI:percutaneous coronary intervention; IVUS: intravascular ultrasound; MI: myocardial infarction; TVR: target vessel revascularization. | | | |  |
|  |  |  |  |  |
|  |  |  |  |  |
