# Supplementary material for: A comparison of long‐term clinical outcomes between percutaneous coronary intervention (PCI) and medical therapy in patients with chronic total occlusion in noninfarct‐related artery after PCI of acute myocardial infarction
Source: Clin Cardiol. 2022 Jan 6;45(1):136–44. doi: 10.1002/clc.23771 (PMC8799053; doi:10.1002/clc.23771)
Supplement: Supplementary file 7 — Supporting information. [file CLC-45-136-s002.docx]

Figurementary Figure 1. Subgroup analysis in ITT population for the primary endpoint. PCI: percutaneous coronary intervention; MT: medical therapy; STEMI:ST-segment elevation myocardial infarction; NSTEMI: non-ST segment elevation myocardial infarction; LVEF: left ventricular ejection fraction; IRA: infarct-related artery; LAD: left anterior descending coronary artery; CTO: chronic total occlusion; ITT: intention-to-treat.

Figurementary Figure 2. Subgroup analysis in PP population for the primary endpoint. s-PCI: successful percutaneous coronary intervention; o-CTO: occluded chronic total occlusion; STEMI:ST-segment elevation myocardial infarction; NSTEMI: non-ST segment elevation myocardial infarction; LVEF: left ventricular ejection fraction; IRA: infarct-related artery; LAD: left anterior descending coronary artery; PP: per-protocol.
